# Metagenomic potential for and diversity of N‐cycle driving microorganisms in the Bothnian Sea sediment

**DOI:** 10.1002/mbo3.475

**Published:** 2017-05-23

**Authors:** Olivia Rasigraf, Julia Schmitt, Mike S. M. Jetten, Claudia Lüke

**Affiliations:** ^1^ Department of Microbiology IWWR Radboud University Nijmegen Nijmegen Netherlands; ^2^ DVGW‐Forschungsstelle TUHH Hamburg University of Technology Hamburg Germany; ^3^ Department of Biotechnology Delft University of Technology Delft Netherlands; ^4^ Soehngen Institute of Anaerobic Microbiology Nijmegen Netherlands

**Keywords:** anammox, Bothnian Sea, denitrification, N‐cycle, sediment

## Abstract

The biological nitrogen cycle is driven by a plethora of reactions transforming nitrogen compounds between various redox states. Here, we investigated the metagenomic potential for nitrogen cycle of the in situ microbial community in an oligotrophic, brackish environment of the Bothnian Sea sediment. Total DNA from three sediment depths was isolated and sequenced. The characterization of the total community was performed based on 16S rRNA gene inventory using SILVA database as reference. The diversity of diagnostic functional genes coding for nitrate reductases (*napA*;*narG*), nitrite:nitrate oxidoreductase (*nxrA*), nitrite reductases (*nirK*;*nirS*;*nrfA*), nitric oxide reductase (*nor*), nitrous oxide reductase (*nosZ*), hydrazine synthase (*hzsA*), ammonia monooxygenase (*amoA*), hydroxylamine oxidoreductase (*hao*), and nitrogenase (*nifH*) was analyzed by blastx against curated reference databases. In addition, Polymerase chain reaction (PCR)‐based amplification was performed on the *hzsA* gene of anammox bacteria. Our results reveal high genomic potential for full denitrification to N_2_, but minor importance of anaerobic ammonium oxidation and dissimilatory nitrite reduction to ammonium. Genomic potential for aerobic ammonia oxidation was dominated by *Thaumarchaeota*. A higher diversity of anammox bacteria was detected in metagenomes than with PCR‐based technique. The results reveal the importance of various N‐cycle driving processes and highlight the advantage of metagenomics in detection of novel microbial key players.

## 1 INTRODUCTION

Baltic Sea is a brackish basin which has been heavily impacted by eutrophication in the past decades (Jäntti & Hietanen, 2012). Intense agriculture and increases in populations have led to elevated input of reactive nitrogen and phosphorous via the riverine input. This eventually resulted in increased productivity, degradation, and expansion of hypoxic waters in wide areas of the Baltic Sea (Zillén, Conley, Andrén, Andrén, & Björck, 2008).

Counteractive to the external input of reactive nitrogen are microbial processes of denitrification and anaerobic ammonium oxidation (anammox) which convert it back into N_2_ and remove it from the system. They occur in both hypoxic water column and sediments, the latter is, however, of particular importance for N cycling as sediments represent hot‐spots of microbial activity due to excess availability of organic matter. The solid matrix of sediments limits the diffusion of substrates and so, through biotic and abiotic reactions, redox gradients establish and spatially separate aerobic and anaerobic metabolisms (Joye & Anderson, 2008).

In a stark contrast, Bothnian Sea which is located in the northern part on the Baltic Sea, has been affected by eutrophication to a far lesser degree and is considered as an oligotrophic ecosystem (Lundberg, Jakobsson, & Bonsdorff, 2009). This was mainly attributed to physical factors such as sea bed topography, weak stratification due to low salinity, and water exchange dynamics (Lundberg et al., 2009). The main input of organic matter into the Bothnian Sea was calculated to be riverine and from occasional intrusions of eutrophic waters from the Baltic Proper (Algesten et al., 2006).

Due to substantial differences in eutrophication status within the Baltic Sea basin, the reactive nitrogen balance and associated microbial processes are expected to differ too. Detailed studies have been performed on sediments of all major arms of the Baltic Sea with emphasis on denitrification, anammox, and dissimilatory nitrate reduction to ammonium (DNRA) (Deutsch, Forster, Wilhelm, Dippner, & Voss, 2010; Hietanen, 2007; Jäntti & Hietanen, 2012; Jäntti, Stange, Leskinen, & Hietanen, 2011; Stockenberg & Johnstone, 1997). The contribution of each of these processes to N‐oxide conversion is of particular importance as it would determine how much reactive nitrogen is being removed from the ecosystem (as N_2_ gas via denitrification and anammox) or recycled into ammonium (via DNRA). Studies on highly eutrophic Gulf of Finland sediments revealed seasonal activity variability for anammox, DNRA, nitrification, and denitrification, with the latter two being closely interdependent (Deutsch et al., 2010; Jäntti et al., 2011). Denitrification was shown to be the dominant N‐oxide sink with anammox contribution under 20% (Hietanen, 2007; Jäntti et al., 2011). However, in events of persistent bottom water anoxia, nitrification is affected by low O_2_ and DNRA begins to dominate over denitrification in N‐oxide reduction (Jäntti & Hietanen, 2012). Also in sediments of other parts of the Baltic Sea, including Baltic Proper and Gulf of Bothnia, denitrification was shown to be the dominant N‐oxide sink (Bonaglia et al., 2016; Deutsch et al., 2010; Stockenberg & Johnstone, 1997). The latest study on in situ N‐cycle activities in brackish sediments of the Bothnian Bay and Bothnian Sea showed that total rates of denitrification were lower than in eutrophic sediments of the southern Baltic Sea basin with anammox contributing at some sites up to 26% in N‐oxide reduction (Bonaglia et al., 2016). Interestingly, DNRA seemed to be of major importance at a coastal shallow, oligotrophic site in the Bothnian Bay, but was not measurable at an offshore site in the Bothnian Sea (Bonaglia et al., 2016).

Despite extensive research on N‐cycle sediment activities, the responsible microbial communities remain largely unknown. Most molecular studies have focused separately either on the diversity of 16S rRNA or selected functional gene biomarkers (Edlund, Hårdeman, Jansson, & Sjöling, 2008; Falk et al., 2007; Glaubitz et al., 2009; Grote, Jost, Labrenz, Herndl, & Jürgens, 2008). A recent study investigated microbial community with emphasis on N‐cycle over a stratified redox gradient in the deepest and highly eutrophic part of the Baltic Sea by a metagenomics approach (Thureborn et al., 2013). Based on functional gene analysis, this site exhibited high potential for autotrophic sulfur‐based denitrification by ε‐Proteobacteria in possible syntrophy with ammonia‐oxidizing *Thaumarchaeota* (Thureborn et al., 2013). Similar trends have been observed at other eutrophic, anoxic sites throughout the Baltic Sea basin (Glaubitz et al., 2009; Grote et al., 2008).

Most of the molecular analyses have, however, focused on eutrophic areas of the Baltic Sea and very little is known about the microbial community composition in oligotrophic sediments of the Bothnian Sea. Moreover, despite the existing activity studies, the underlying functional N‐cycle potential of these sediments remains unknown and thus comparisons to other parts of the Baltic Sea are not possible.

**Figure 1:**
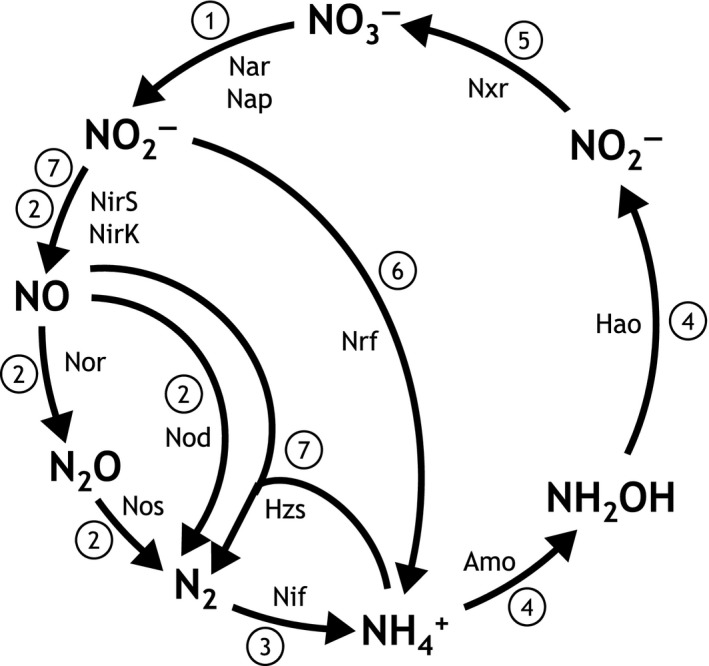
Biological N‐cycle illustrating major metabolic processes (number coded) with associated known key enzymes. Number‐coded metabolic processes: 1, nitrate reduction; 2, denitrification; 3, nitrogen fixation; 4, aerobic ammonia oxidation; 5, aerobic nitrite oxidation; 4+5, comammox; 6, dissimilatory nitrite reduction to ammonium (DNRA); 7, anaerobic ammonia oxidation. Nar/Nap, dissimilatory nitrate reductase; NirK/NirS, dissimilatory NO‐forming nitrite reductase; Nor, nitric oxide reductase; Nod; nitric oxide dismutase; Nos, nitrous oxide reductase; Nif, nitrogenase; Amo, ammonia monooxygenase; Hao, hydroxylamine oxidoreductase; Nxr, nitrite:nitrate oxidoreductase; Nrf, dissimilatory ammonia‐forming nitrite reductase; Hzs, hydrazine synthase

In this study we investigated the phylogenetic composition and metagenomic potential of the in situ microbial community with respect to the biological N‐cycle in the Bothnian Sea sediment at three depths. Curated datasets of the diagnostic N‐cycle proteins (Figure 1) were used to estimate the abundance and diversity of the various reactions with specific emphasis on the anammox process.

## 2 MATERIALS & METHODS

### 2.1 Sampling site and core processing

**Figure 2:**
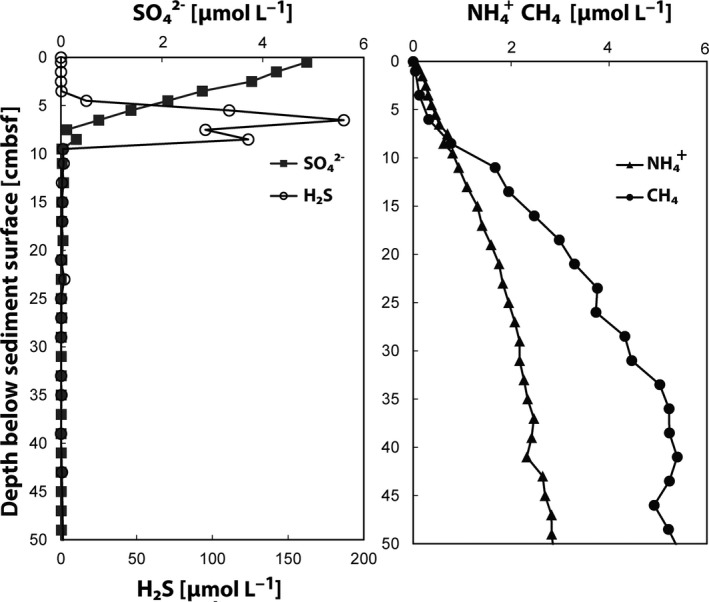
Geochemical profiles for site US5B in the Bothnian Sea sediment (data were published previously in Egger et al., 2015)

Sediment cores were taken in the Bothnian Sea at sampling site US5B during the R/V *Aranda* cruise in August 2012 (62°35.17′N, 19°58.13′E, location, sampling procedure, and core storage are described in Egger et al., 2015). Biogeochemical parameters were measured either onboard or later at the laboratory as previously described (Egger et al., 2015). Key biogeochemical parameters are shown in Figure 2. One sediment core dedicated to molecular analysis was analyzed in this study. It was sliced under aerobic conditions in intervals of 2.5 cm, immediately frozen in liquid nitrogen and stored at −80°C.

### 2.2 DNA isolation and sequencing

Total DNA was extracted from each homogenized sediment core slice with the PowerSoil Total RNA Isolation Kit with DNA Elution Accessory kit (MoBio). For each extraction, 2 g of sediment material was used according to manufacturer's instructions. The quality and quantity of isolated DNA was accessed with NanoDrop 1000 (Thermo Scientific). For each analyzed depth (0–2.5, 5–12.5, and 30–35 cm below the surface (cmbsf)), the isolated DNA was pooled in equimolar concentrations, if necessary. DNA samples were stored at −20°C until further metagenomic library preparation.

Metagenomic library preparation was performed with IonXpress^™^ Plus gDNA Fragment Library kit (Ion Torrent^™^ platform, Life technologies) following the manufacturer's instructions. The initial shearing of DNA was performed by ultrasonication (Bioruptor^®^, Diagenode) for 7 min. The quality and quantity of DNA were assessed with the Bioanalyzer 2100 during the library preparation procedure. Sequencing was performed with the Ion PGM^™^ system (Ion Torrent^™^ platform, Life technologies).

### 2.3 Polymerase chain reaction

Polymerase chain reaction (PCR) reactions were performed to amplify the *hzsA* gene specific to anammox bacteria in samples from 0 to 25 cmbsf on each 2.5‐cm interval sample individually. PCR reaction was composed as previously described (Harhangi et al., 2012). Following primer pairs were used: hzsA_757F and hzsA_1829R to cover the diversity of known freshwater anammox bacteria, and hzsA_757F Scalindua and hzsA_1829R Scalindua to cover known marine anammox bacteria (Harhangi et al., 2012). PCR was performed in a thermocycler (Professional thermal cycler, Biometra, Jena) with following parameters: initial denaturation for 4 min at 94°C, followed by 30 cycles of denaturation for 1 min at 94°C, primer annealing for 1 min at 43–56°C (parallel PCR reactions were performed at different annealing temperatures), elongation for 2 min at 72°C, and final elongation for 10 min at 72°C. PCR products were checked with gel electrophoresis. Due to low final concentration of PCR products, a seminested PCR was performed with hzsA_1600F Scalindua and hzsA_1829R Scalindua primers (Harhangi et al., 2012). PCR reaction products for each depth sample were pooled and subjected to gel electrophoresis. Bands of correct size were cut and purified from gels with the Gene Jet Gel extraction kit (Thermo Scientific, Waltham) following manufacturer's instructions. A seminested PCR was performed under following conditions: initial denaturation for 4 min at 94°C, followed by 30 cycles of denaturation for 1 min at 94°C, primer annealing for 1 min at 56°, elongation for 2 min at 72°C, and final elongation for 10 min at 72°C. Ligation, transformation, and cloning of PCR products were performed as previously described (Harhangi et al., 2012).

### 2.4 Metagenome analysis

Raw sequence data were trimmed to >100nt length with CLC Bio Genomics Workbench 7.0.3 (CLC Bio, Qiagen) resulting in the following read numbers: 3,966,999 (average length 289 nt) for 0–2.5 cmbsf, 3,615,605 (average length 296 nt) for 5–12.5 cmbsf, and 3,225,702 (average length 291 nt) for 30–35 cmbsf. The phylogenetic characterization of in situ microbial community was performed based on 16S rRNA gene diversity. The raw metagenomic sequence reads were mapped to a reference SSU rRNA gene dataset obtained from the SILVA database (Quast et al., 2013) (RefNR99 dataset, release 115) using CLC Genomics Workbench with the following mapping parameters: mismatch cost 2, insertion cost 3, deletion cost 3, length fraction 0.5, and similarity fraction 0.8. The reads that mapped to SSU rRNA reference gene set were extracted and used for subsequent nucleotide blast analysis against the same SSU reference dataset (e‐value cut‐off 10E‐6). Significant hits were extracted and aligned using the SINA Aligner (Pruesse, Peplies, & Glöckner, 2012). The aligned SSU rRNA reads were then imported into ARB and added to the existing Neighbor Joining reference phylogenetic tree with the QuickAdd option (Ludwig et al., 2004).

The analysis of diagnostic genes encoding key enzymes involved in the nitrogen cycle (Figure 1) was performed with the analysis pipeline described previously (Lüke, Speth, Kox, Villanueva, & Jetten, 2016). Metagenomes were blasted against manually curated functional gene databases (e‐value cutoff 10E‐6), the reads extracted and then reblasted against the nonredundant protein database (NCBI nr). Both blast outputs were plotted based on the resulting bit scores resulting in a linear bit score ratio plot. This method was used in order to sort out false positives, but still keep divergent gene reads with a significant hit against the analyzed protein (Lüke et al., 2016). For each analyzed gene, a different threshold bit score ratio (based on manual checking) was used in order to sort out false‐positive hits. Cut‐off bit score ratios used in this study are summarized in Table S1. After the curation procedure, the extracted reads were again blasted against the nonredundant protein database and the blast output was imported into MEGAN (Huson, Mitra, Ruscheweyh, Weber, & Schuster, 2011) for further phylogenetic characterization and quantification. Following settings were used: min score 50, max expected 0.01, top percent 1, max support percent 0.0, min support 1, LCA percent 50, and min complexity 0. For quantitative comparison between genes and metagenomes, the analyzed gene reads were normalized to metagenome size and average gene length according to the following formula: normalized read count (*nrc*) = (gene read count*100,00,00,000)/(total metagenome read count*average gene length [nt]). The differentiation between *napA* and *narG* genes was performed in MEGAN using the KEGG orthology, and the extracted subsets were then classified separately.

The curated functional gene databases used for blast analysis were published previously (Lüke et al., 2016).

The raw reads of metagenome sequencing described in this study (OAZ, SMTZ and MZ) have been deposited to the European Nucleotide Archive, with study accession number PRJEB20768.

## 3 RESULTS AND DISCUSSION

### 3.1 16S rRNA gene‐based phylogenetic community composition

**Figure 3:**
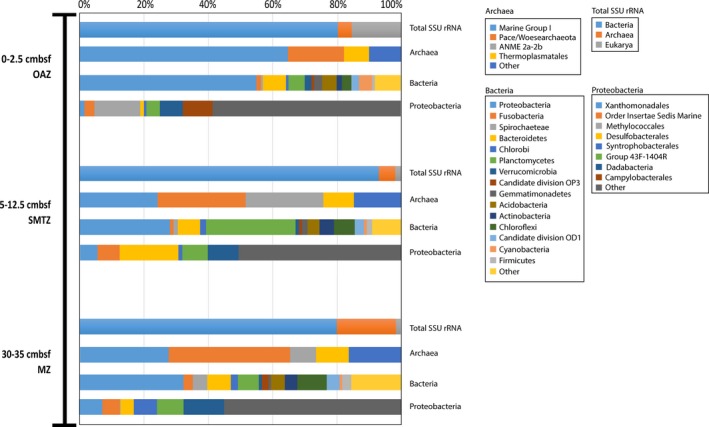
16S rRNA gene distribution (normalized values) over the three analyzed depths in the Bothnian Sea sediment profile. Bacterial 16S rRNA is shown for groups with abundances over 2%, archaeal and proteobacterial over 5%. ANME, Anaerobic Methanotrophic Archaea; OAZ, oxic/anoxic interface zone; SMTZ, sulfate methane transition zone; MZ, methanic zone

The microbial community composition over the sediment core from the site US5B in the Bothnian Sea was analyzed with respect to 16S rRNA diversity and N‐cycle‐related diagnostic genes from three different depths: oxic/anoxic interface zone (OAZ), sulfate methane transition zone (SMTZ), and methanic zone (MZ) (Figure 3). Total SSU rRNA gene reads from each analyzed sediment sample comprised approximately 5%–6% of total raw reads in respective metagenomes. The majority was assigned to bacteria (80% in OAZ, 93% in SMTZ, and 80% in MZ) with archaea contributing 4% in OAZ, 5% in SMTZ, and 18% in MZ.

### 3.2 Sediment in situ bacterial composition

The most abundant bacterial phylum in OAZ and MZ was *Proteobacteria* with 55% and 32%, respectively. Other abundant phyla were comprised by *Bacteroidetes* (7% in both samples), *Planctomycetes* (5% in OAZ and 6% in MZ), and *Chloroflexi* (3% in OAZ and 9% in MZ). In SMTZ, *Proteobacteria* were as abundant as *Planctomycetes* (28% each), followed by *Bacteroidetes* (7%) and *Chloroflexi* (6%). The proportional distribution of the most abundant archaeal, bacterial, and proteobacterial groups in all samples is shown in Figure 3.

The distribution within the proteobacterial population varied substantially between all depth samples. Whereas the most dominant groups in OAZ were comprised by *Methylococcales* (14%), *Campylobacterales* (9%), and unclassified group Sh765B‐TzT‐29 (7%), the SMTZ was dominated by *Desulfobacterales* (18%), group Sh765B‐TzT‐29 (10%), group 43F‐1404R (8%), and Order Insertae Sedis/Family Insertae Sedis/Marine (7%). The MZ was dominated by group Sh765B‐TzT‐29 (13%), group 43F‐1404R (8%), *Syntrophobacterales* (7%), and *Xanthomonadales* (7%).

The occurrence of *Methylococcales* in the upper depth corresponded to availability of both methane and oxygen in this depth which is essential for the methanotrophic lifestyle of this group. *Campylobacterales*, the second most abundant proteobacterial group in OAZ, comprised the genera *Sulfurimonas* and *Sulfurovum* belonging to the family *Helicobacteraceae*. Both *Sulfurovum* and *Sulfurimonas* spp. are commonly found in marine environments, they respire oxygen or nitrate with reduced sulfur species as electron donors (Inagaki, Takai, Kobayashi, Nealson, & Horikoshi, 2003; Inagaki, Takai, Nealson, & Horikoshi, 2004; Zhang, Zhang, Shao, & Fang, 2009). These findings are corroborated by profiles of nitrate and oxygen which were available only within the uppermost centimeter (cmbsf), but were not measurable below these depths (Egger et al., 2015; Slomp et al., 2013). Moreover, reduced sulfur‐fueled denitrification potentially coupled to archaeal ammonia oxidation was found to be a dominant process in the eutrophic parts of the Baltic Sea (Glaubitz et al., 2009; Thureborn et al., 2013).

The coupling between biogeochemistry and community structure was also apparent in the SMTZ. Here, the dominance of *Desulfobacterales* corresponded to the availability of both sulfate and methane, which are removed during anaerobic methane oxidation by methanotrophic archaea (ANME) and sulfate‐reducing bacteria (SRB). *Desulfobacterales* are often observed as the SRB partner of ANME (Knittel, Lösekann, Boetius, Kort, & Amann, 2005; Michaelis et al., 2002; Siegert, Krüger, Teichert, Wiedicke, & Schippers, 2011).

Interestingly, group Sh765B‐TzT‐29 falling within δ‐Proteobacteria (according to the applied SILVA taxonomy) was found to be abundant in all depths. After sequencing a single‐cell genome from one of the representatives of this group, a recent study reclassified it as *Candidatus* Dadabacteria which represents a novel phylum rather than a branch within δ‐Proteobacteria (Hug et al., 2016). The available genome information provided evidence for the use of organic carbon with complete glycolysis and TCA cycle pathways (Hug et al., 2016). 16S rRNA gene sequences from this group have been found in various anoxic environments and it was discussed that it might be involved in sulfur cycling (Hietanen, 2007; Jäntti & Hietanen, 2012; Siegert et al., 2011), nitrate reduction, or DNRA (Hug et al., 2016).

Also a single‐cell genome from the δ‐proteobacterial group 43F‐1404R, abundant in both SMTZ and MZ, has been recently sequenced and provided more clues about its physiological potential (Hug et al., 2016). Members of this group seem to be heterotrophs with canonical electron transport chain, capacity for nitrate reduction, and nitrite reduction to ammonium (Hug et al., 2016). 16S rRNA gene sequences from this group have been detected previously in marine sediments with active sulfur cycling (Asami, Aida, & Watanabe, 2005), marine hydrothermal field sediments (Kato et al., 2009), and paddy soils (Itoh et al., 2013).

In MZ, a relative increase in abundance of *Syntrophobacterales* was observed. Members of this order are closely related to SRB (McInerney et al., 2008). They were shown to be metabolically flexible and depending on environmental conditions and metabolic partners able to perform sulfate respiration or fermentation (Plugge, Zhang, Scholten, & Stams, 2011). *Syntrophobacterales* are often found in syntrophic partnerships with hydrogen‐consuming organisms in anoxic methanogenic environments (Lueders, Pommerenke, & Friedrich, 2004; Stams & Plugge, 2009). An abundant methanogen population would potentially act as a hydrogen sink in this depth.

The *Planctomycete* population differed substantially within the sediment transect with a remarkable abundance of *Brocadiales*‐related sequences (65% of all *Planctomycete* reads) in SMTZ versus 5% in OAZ and none in MZ. Members of *Brocadiales* are known for their capacity to oxidize ammonium with nitrite in the absence of oxygen (anammox) (Strous et al., 2006). All gene sequences (including functional) associated with anammox bacteria peaked in the putative SMTZ zone despite the apparent absence of available nitrite in this zone.

### 3.3 Archaeal in situ sediment composition

The most dominant groups within the archaeal population at all depths were the thaumarchaeal Marine Group I (MG‐I), Deep Sea Hydrothermal Vent Group 6 (DSHVG‐6), *Thermoplasmatales,* and the ANME‐2.

Group MG‐I comprised 65% of all archaeal 16S rRNA gene reads in OAZ, whereas in SMTZ and MZ its relative abundance was with 24% and 28% substantially lower, respectively. Previous studies have shown that the representatives of this group are capable of aerobic oxidation of ammonia (AOA) (Stahl & de la Torre, 2012). Furthermore, MG‐I seems to be dominant among archaea in various marine water and sediment habitats underlining its importance for biogeochemical element cycling (Dang, et al., 2010b; Dang, et al., 2013a; DeLong, 1992; Galand, Casamayor, Kirchman, Potvin, & Lovejoy, 2009). Although most gene reads belonging to MG‐I were detected in OAZ where both oxygen and nitrate co‐occurred, their relatively high abundance in deeper anoxic layers was puzzling. Similar observations were reported previously from deep oligotrophic sediment subsurface (Inagaki et al., 2006; Sørensen, Lauer, & Teske, 2004; Teske, 2006), estuarine and marine sediments of the South China Sea (Dang et al., 2013a), and deep‐sea methane seep environments in the Okhotsk Sea (Dang, et al., 2010b). The metabolic function of these deep‐sediment MG‐I group archaea remains unclear. A positive correlation between the occurrence of archaeal *amoA* and sediment organic matter content led to a speculation that they might possess a heterotrophic lifestyle (Dang, et al., 2010b).

Group DSHVG‐6 was second most abundant in the Bothnian Sea sediment and its relative abundance increased with depth (17% in OAZ, 27% in SMTZ, and 38% in MZ). Recent metagenomic efforts have obtained several single‐cell genomes of this group originating from anoxic aquifers, and reclassified it into separate *Woesearchaeota* and *Pacearchaeota* sister phyla (Castelle et al., 2015). The available genomic data point to anaerobic fermentative and/or symbiosis‐based lifestyles without a consistent metabolic signal within the phylum‐level radiations (Castelle et al., 2015). Interestingly, some member genomes encoded archaeal type III ribulose 1,5‐bisphosphate carboxylase/oxygenase (RuBisCO) indicating a possible function in the nucleotide salvage pathway (Castelle et al., 2015). 16S rRNA gene sequences of these archaea have been detected in various marine and freshwater environments. Investigations of methane seep sediments from Nankai Through have revealed similar trends in archaeal populations with groups MG‐I and *Pace‐/Woesearchaeota* being the most dominant and showing a decrease in MG‐I and increase in *Pace‐/Woesearchaeota* abundance with depth (Nunoura et al., 2012).

*Thermoplasmatales* archaea were found in relatively constant abundance (8%–10%) at all depths over the sediment transect. The sequences clustered within five groups (SILVA phylogeny): ASC21, AMOS1A‐4113‐D04, Marine Group II, Terrestrial Miscellaneous Group (TMEG), and 2B5. Their metabolism remains unknown, but related 16S rRNA sequences have been detected in methane seeps of the North Sea (Wegener et al., 2008), subseafloor sediments in gas hydrate area, oxygen minimum zone in Pacific (unpublished), methanogenic estuarine sediments in Orikasa River (Kaku, Ueki, Ueki, & Watanabe, 2005), and deep‐sea anoxic sediments of the Okhotsk Sea (Dang, Luan, Zhao, & Li, 2009; Dang, et al., 2010b).

ANME archaea were only detected in SMTZ (24%) and MZ (8%) which is in correspondence to methane availability in these depths. These archaea are involved in anaerobic methane oxidation in cooperation with SRB from the order *Desulfobacterales* (Knittel et al., 2005).

### 3.4 N‐cycle diagnostic gene analysis

**Figure 4:**
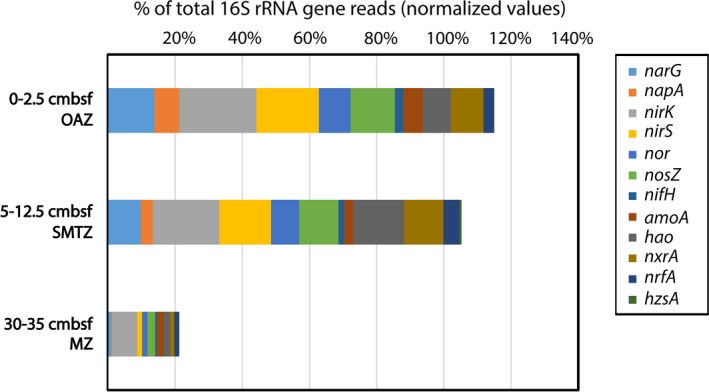
Proportional distribution of N‐cycle related normalized gene reads in relation to total 16S rRNA normalized reads along the analyzed depth profile at site US5B in the Bothnian Sea. cmbsf, cm below sediment surface; OAZ, oxic/anoxic interface zone; SMTZ, sulfate methane transition zone; MZ, methanic zone

The combined functional gene analysis was performed in order to assess the metabolic potential for various N‐cycle processes and to relate it to the population diversity based on 16S rRNA gene analysis at the site US5B. The overview of the proportional distribution of N‐cycle‐related functional gene reads in relation to normalized total 16S rRNA over the depth profile is shown in Figure 4. Numbers of reads mentioned in the text refer to normalized read counts (*nrc*) (Table S2).

### 3.5 Dissimilatory nitrate reduction: nitrate reductase (*narG/napA*)

The nitrate‐reducing community harboring *narG* showed a clear stratification within the sediment transect. Most reads (55) were found in OAZ, where oxygen and nitrogen oxides were still available for respiration. Roughly half of all reads were assigned to phylum *Proteobacteria* with the most dominant groups belonging to *Methylococcales* (3.3), *Desulfuromonadales* (3.5), and *Rhodocyclales* (5.3). Other dominant *narG*‐containing groups were assigned to candidate division OP3 (*Omnitrophicae*, 9.9) and *Haloarchaea* (3.4). In SMTZ (34), the major groups containing *narG* were comprised by *Rhodocyclales* (4.7), *Desulfobacterales* (2.3), *Deinococcales/Thermales* group (1.8), candidate division OP3 (*Omnitrophicae*, 3.6), and *Haloarchaea* (2.9). Notably, the change in biogeochemical parameters toward the absence of oxygen and dominance of sulfur cycle in this zone was accompanied by the shift in *narG*‐harboring community toward sulfate‐reducing bacteria (*Desulfobacterales*) and decline in methane‐oxidizing bacteria (*Methylococcales*) and iron/iron/sulfur‐reducing bacteria (*Desulfuromonadales*). The deeper MZ layer was characterized by a stark decrease in overall *narG* read numbers (3) most of which were assigned to candidate division OP3 (*Omnitrophicae*, 0.9) with the remaining reads being distributed among *Proteobacteria*. These results were congruent with all other observations and indicated the low potential and need for nitrate reduction in this depth.

Conspicuous was the dominance of candidate division OP3 within the *narG*‐harboring community in all depths. There are so far no cultured representatives from this group and their metabolic potential remains elusive. 16S rRNA gene information revealed that candidate division OP3 belongs to the *Planctomycetes/Verrucomicrobia/Clamydiae* (PVC) superphylum and it was suggested that members of this group are most likely anaerobes thriving in marine sediments, lakes, and aquifers (Glöckner et al., 2010; Ragon, Van Driessche, Garcia Ruiz, Moreira, & Lopez‐Garcia, 2013). Currently available genome information obtained from single cells and a waste water treatment plant for members of this group, provisionally named *Omnitrophica*, revealed the presence of genes coding for respiratory nitrate reductase, heme/copper‐type cytochrome/quinol oxidases, and nitric oxide reductases (Speth, In ‘t Zandt, Guerrero‐Cruz, Dutilh, Jetten, 2016). However, complete gene sets encoding the full denitrification pathway could not be detected. Another recent study found strong correlations between the occurrence of candidate division OP3 16S rRNA genes and the content of oxidized iron minerals at the discharge zone of an intertidal aquifer, speculating on the possible involvement of some members of this group in iron cycling (McAllister et al., 2015). The iron‐rich sediments of the Bothnian Sea might thus provide a suitable habitat for members of this group.

*napA* gene reads were less abundant than *narG*. The distribution followed the pattern of *narG* with most reads detected in OAZ (30), where most abundant groups belonged to *Flavobacteriia* (1.9), *Campylobacterales* (4.1), and *Alteromonadales* (3.9). The distribution of *napA* in SMTZ (12) was more evenly spread with most abundant groups belonging to *Planctomycetes* (1.1), *Burkholderiales* (1.1), *Desulfobacterales* (0.9), *Desulfuromonadales* (0.9), and *Campylobacterales* (1.1). In MZ, *napA* was with one detected read only of minor importance consistent with the finding on *narG* and other N‐cycle genes.

### 3.6 Denitrification/anammox: Dissimilatory NO‐forming nitrite reductase *(nirS;nirK)*

The distribution of both *nirK* and *nirS* gene reads followed the same trend as for other N‐cycle‐related genes with decreasing abundance with increasing depth. A large proportion (39.4) of detected *nirK* reads in OAZ (total of 92) was assigned to *Thaumarchaeota*. Next abundant groups were assigned to *Methylococcales* (4.1) and *Rhizobiales* (3.4). The most abundant *nirK* groups in SMTZ (total of 68) belonged to *Thaumarchaeota* (11.3), *Actinobacteria* (7), and *Rhizobiales* (2.7), indicating a clear community shift with the increasing depth. Interestingly, deeper in the sediment transect (MZ), the thaumarchaeal *nirK* seemed to increase in both relative abundance and the total *nrc* value (14.5) in comparison to SMTZ, despite the observation of total group MG‐I 16S rRNA gene reads being significantly lower in abundance in this depth.

The available genome information of AOA has confirmed the presence of multiple gene copies of multicopper oxidoreductase‐type nitrite reductases, a trait which seems to be highly conserved among AOA (Lund, Smith, & Francis, 2012). This led to a conclusion that AOA might be able to perform nitrifier denitrification, and findings of marine N_2_O produced by AOA further corroborated this idea (Santoro, Buchwald, McIlvin, & Casciotti, 2011). Recent findings, however, point to the inability of *Thaumarchaeota* to enzymatic production of N_2_O via NO in the classical process of nitrifier denitrification (Kozlowski, Stieglmeier, Schleper, Klotz, & Stein, 2016), pointing to a different route.

The physiological role of AOA NirK remains unclear, it might be involved in detoxification of nitrite or use of nitrite as the alternative electron acceptor to oxygen under hypoxia (Walker et al., 2010). Environmental studies have revealed wide distribution of thaumarchaeal *nirK* genes in marine water columns and sediments (Lund et al., 2012; Venter et al., 2004; Yakimov et al., 2011), also indicating distinct communities between water columns and sediments (Lund et al., 2012). The calculated ratios of *nirK* to group MG‐I 16S rRNA showed a decrease with increasing depth (3.5 in OAZ, 2.7 in SMTZ, and 0.9 in MZ).

*Proteobacteria* was the most dominant bacterial phylum harboring *nirS*‐like genes in OAZ (36.4), SMTZ (24), and MZ (1.4). Order *Methylococcales* represented with 8.1, the most abundant *nirS*‐harboring group in OAZ. This finding corresponded to widespread occurrence of *Methylococcales* bacteria in this depth as inferred from 16S rRNA and findings of other genes of the denitrification pathway (e.g., *narG*,* nor, nirK*). Available genome data of methane‐oxidizing bacteria confirm widespread presence of genes involved in denitrification and their contribution to N_2_O production (Stein & Klotz, 2011). Recent work has shown that some methanotrophic bacteria encode and express denitrification genes when exposed to nitrate and are able to link the reduction in nitrate to the oxidation of methane under hypoxia in a bioenergetically favorable manner (Kits, Klotz, & Stein, 2015).

### 3.7 Denitrification: Dissimilatory nitric oxide reductase (*nor*)/nitric oxide dismutase (*nod*)

*nor*‐like genes were detected in all depths over the sediment transect with decreasing abundance with increasing depth (38 in OAZ, 29 in SMTZ, and 5 in MZ). In OAZ, no clear dominance of any taxonomic group could be observed. Here, among the most abundant groups to which the *nor*‐like gene reads were assigned were *Spirochaetales* (2.2), *Methylococcales* (2.4), *Desulfuromonadales* (2.2), *Myxococcales* (2.9), *Burkholderiales* (2.8), and *Planctomycetes* (2.1). Notably, large proportion of *nor‐*like reads showed nearest identity to *Flavobacteriia* (3.4) and strain HdN1 (2.9). Several *nor*‐like reads from the *Flavobacteriia* order revealed highest identity to *Muricauda ruestringensis* and other organisms containing alternative *nor*‐like genes with sequence features found in Nod proteins. Also, sequences resembling nearest identity to strain HdN1 *nod* sequence pointed to an abundant population of bacteria‐containing Nod‐like proteins. However, no 16S rRNA genes affiliated with either *M. oxyfera* or strain HdN1 could be detected pointing to novel *nod*‐like gene‐harboring bacterial groups.

The observation of abundant *nod*‐like gene reads would be congruent with biogeochemical data indicating the presence of methane and nitrogen oxides in OAZ for N‐AOM. However, an alternative Nod‐catalyzed metabolism might be possible. Also, similar *nor*‐containing phylogenetic groups were identified in SMTZ; however, the abundances differed to OAZ. In particular, the abundance of *nor*‐like *nrc* assigned to *Desulfuromonadales* and *Flavobacteriia* increased to 4, respectively. The abundance of *nod*‐like gene‐containing strain HdN1‐like population remained similar (2.2). This finding showed that despite the absence of nitrogen oxides in SMTZ, *nod‐*like genes were still relatively abundant. This pointed to either a nonactive population of bacteria containing Nod proteins or an active population using Nod‐like proteins to perform an alternative metabolism.

### 3.8 Denitrification: Nitrous oxide reductase (*nosZ*)

Our metagenome analysis revealed high abundance of *nosZ‐*encoding gene reads (53 in OAZ, 40 in SMTZ, and 8 in MZ, respectively) which was approximately within the same range as the abundance of *narG*‐ and *nirS*‐encoding gene reads. Most dominant phylum harboring *nosZ* gene in OAZ was assigned to *Bacteroidetes* with *Flavobacteriia* (12), *Cytophagales* (3.5), and Bacteroidetes Order Insertae II Sedis (2.5) groups being the most dominant. Similar bacterial groups were dominating the SMTZ, however, the abundances changed. *nosZ*‐like *nrc* assigned to *Flavobacteriia* decreased to 7.1 and those to *Myxococcales* increased to 1.8. In MZ, *Flavobacteriia* was with *nrc* of 2.1 the most dominant *nosZ*‐harboring group.

These results pointed to widespread capacity for nitrous oxide reduction in the Bothnian Sea sediment along the whole sediment transect with molecular nitrogen being the most likely product of denitrification.

### 3.9 Nitrogen fixation: Nitrogenase (*nifH*)

Analysis for nitrogen fixation potential in the sediment transect revealed low abundance of *nifH* gene reads in all sediment depths (10 in OAZ, 6 in SMTZ, and 2 in MZ) as well as clear differences in microbial populations responsible for the process. The majority of detected *nifH* gene reads in OAZ was assigned to *Methylococcales* (7.3) with *Methylobacter* as the most dominant genus (up to 100% identity on protein level). This dominance of *Methylococcales* was congruent with 16S rRNA gene data. The remaining reads were mostly assigned to other γ*‐*Proteobacteria.

The *nifH* inventory in SMTZ revealed a shift in nitrogen fixing population toward *Methanomicrobia* (3.1). The remaining potential nitrogen fixing population was represented by putatively SRB from δ*‐*Proteobacteria, *Nitrospirae* and *Firmicutes*.

The phylogenetic affiliation of *nifH* reads within the sediment transect reflected the dominance of major functional microorganism groups in each particular depth observed from the 16S rRNA analysis.

### 3.10 Aerobic ammonium oxidation: ammonia monooxygenase (*amoA*)

At our sampling site, *amoA* gene sequences were found in all three analyzed depths, with decreasing abundance with increasing sediment depth. The highest abundance was observed in OAZ (23), a sediment zone where oxygen and ammonium still co‐occurred, thus providing substrates for ammonia oxidizers. Taxonomic assignment revealed that the majority of *amoA* reads was assigned to *Thaumarchaeota*, which strongly pointed to their dominance in aerobic ammonia oxidation process in the Bothnian Sea sediment. All archaeal sequences fell within the Marine Group 1.1a. Reads revealed high similarity to sequences found in ecosystems ranging from fully marine over brackish to terrestrial. The closest cultured representatives were *Nitrosopumilus* and *Nitrosoarchaeum* spp. *AmoA* reads were also detected in SMTZ (9) and MZ (6), where electron acceptors other than sulfate or CO_2_ were not available. Most sequences detected in the deeper sediment revealed high similarity to sequences found in marine, estuarine, and freshwater habitats. The analysis of metagenomes for bacterial *amoA* gene reads resulted in two reads in each OAZ and MZ assigned to *amoA*‐like bacterial sequences, respectively. This showed that although bacterial ammonia oxidizers were not completely absent at site US5B, they were probably not of significant importance for N‐cycle transformations at this site.

Based on these observations, it was evident that AOA were not restricted to sediment zones where oxygen was still present, but rather occurred in all analyzed sediment depths. This corresponded to 16S rRNA gene results of MG‐I *Thaumarchaeota* which were detected in all depths and decreased in abundance with increasing sediment depth. The ratio of *amoA* to 16S rRNA of MG‐I was approximately 2 for the upper two depths and 0.3 for MZ. Currently available genomic information of archaeal ammonia oxidizers shows *amoA* and 16S rRNA being single‐copy genes in sequenced genomes of thaumarchaeal ammonium oxidizers. However, several previous studies have reported *amoA*/16S rRNA ratios to be higher than 1 and speculated on several *amoA* copies in AOA genomes (Beman, Popp, & Francis, 2008; Lund et al., 2012; Santoro, Casciotti, & Francis, 2010), which might be the case for novel sedimentary AOA. The occurrence of group MG‐I *Thaumarchaeota* in anoxic sediment layers has been reported previously (Dang, et al., 2010b; Dang et al., 2013a; Jorgensen et al., 2012; Roussel et al., 2009; Sørensen et al., 2004). It has been speculated that electron acceptors other than oxygen might be used for ammonia oxidation in these organisms, or that ammonia monooxygenase might serve a different function than ammonia oxidation (Jorgensen et al., 2012; Mußmann et al., 2011). It has also been shown recently that not all *Thaumarchaeota* are capable of ammonia oxidation, but are metabolically more flexible and can grow with organic nitrogen substrates (Weber, Lehtovirta‐Morley, Prosser, & Gubry‐Rangin, 2015).

### 3.11 Aerobic ammonium oxidation/anammox: hydroxylamine oxidoreductase (*hao*)

*hao*‐like gene reads and its multiheme cytochrome *c* homologs were detected in all depths of the sediment transect; however, unlike other N‐cycle genes, more reads were detected in SMTZ than in OAZ (52 vs. 33). This higher abundance was mostly attributable to the dominance of anammox (order *Brocadiales*) and sulfate‐reducing bacteria (orders *Desulfobacterales* and *Desulfovibrionales*). Based on available genome information, anammox bacteria contain up to 10 divergent *hao*‐like gene paralogs (Kartal, van Niftrik, Keltjens, Op den Camp, & Jetten, 2012; Strous et al., 2006; van de Vossenberg et al., 2013), at least one of which was designated as hydrazine dehydrogenase and shown to be responsible for the oxidation of hydrazine to N_2_ (Maalcke et al., 2016).

This observation was in congruence with 16S rRNA and *hzsA* gene data which all indicated higher abundance of anammox bacteria in SMTZ. The *hao*‐like *nrc* assigned to anammox was 3.9 for OAZ and increased to 8.9 for SMTZ. Blast‐based analysis revealed that the majority (75%) of anammox‐like *hao* gene sequences originating from SMTZ were assigned to *Kuenenia stuttgartiensis* as the closest relative with amino acid identities ranging between 50 and 100%, the remaining reads were assigned to *Scalindua* spp. (14% with identities between 54 and 100%), *Brocadia* spp. (9%, identities between 52 and 70%), and *Jettenia* (2%, 58% identity). In contrast, the majority of reads assigned to anammox in OAZ resembled highest identity to *Scalindua* spp. (65%, accession nr. WP_034410018) with identities ranging between 54 and 100%, the rest was assigned to *Kuenenia* spp. (15%, identities between 47 and 65%), *Brocadia* spp. (7%, identities between 53 and 64%), and *Jettenia* (11%, identities between 76 and 78%). These results pointed to diversity differences in both depths and a presence of a potentially novel anammox genus, an observation which was supported by data derived from 16S rRNA and *hzsA* gene phylogeny. Potentially novel clades of anammox bacteria, deduced from amplified *hzo* gene sequences, have also been reported previously from sediments of the Jiaozhou Bay in China (Dang et al., 2010a). However, as the phylogenetic resolution was limited due to short read length, the interpretation of current data should be treated with care and needs further investigation.

Previous surveys revealed marine ecosystems to be dominated by anammox bacteria of the *Scalindua* genus and freshwater terrestrial habitats by the genera *Kuenenia*,* Brocadia*,* Jettenia,* and *Anammoxoglobus* (Galán et al., 2009; Humbert et al., 2009; Kuypers et al., 2003; Penton, Devol, & Tiedje, 2006). Molecular studies based on amplification of anammox‐specific 16S rRNA and functional genes have reinforced a hypothesis salinity being the major environmental factor shaping the community shifts between the dominance of either *Scalindua* or other anammox genera (Dale, Tobias, & Song, 2009; Hirsch, Long, & Song, 2011), the latter designated as “freshwater” anammox genera. However, physiological studies have shown that members of the genus *Kuenenia* can gradually be adopted to high salt concentrations in a bioreactor system (Kartal et al., 2006), thus indicating that environmental parameters other than salinity might play a role in anammox distribution.

In general, the major proportion of *hao*‐like gene reads in all depths was comprised by δ‐Proteobacteria: 8.9 in OAZ, 18.2 in SMTZ, and 1.4 in MZ, with *Desulfobacterales*,* Desulfovibrionales, Syntrophobacterales,* and *Myxococcales* being the most abundant orders. Due to short read length and thus limited sequence information, accurate function predictions of detected *hao*‐like genes fragments were not possible. However, previous studies have shown several SRB, in particular within the δ‐subdivision, to possess multiheme cytochrome *c* proteins of the C_554_ and other families (Pereira et al., 2011). In SRB, these proteins were speculated to be involved in respiration as in storage of electrons derived from periplasmic hydrogen oxidation (Heidelberg et al., 2004), enzymatic metal reduction (Lovley & Phillips, 1994; Lovley, Roden, Phillips, & Woodward, 1993; Michel, Brugna, Aubert, Bernadac, & Bruschi, 2001), regulation (Pereira et al., 2011), or detoxification (Greene, Hubert, Nemati, Jenneman, & Voordouw, 2003). To our knowledge, there is no evidence for *bona fide* hydroxylamine oxidoreductase proteins in these organisms.

Interestingly, *hao*‐like gene reads affiliated with AOB were detected in low abundance in all analyzed depths (between 0.5 and 1). The AOB‐like *hao* reads were assigned to *Nitrosomonadales* within β‐Proteobacteria. In addition to 16S rRNA and *amoA* gene data, this was another indication of AOB being of low importance in ammonium oxidation at the US5B sampling site.

In contrast to the two upper depths, *hao*‐like genes were of much less importance in MZ depth (6). This trend followed other N‐cycle genes, which pointed to a much lower importance of nitrogen cycling in this depth.

### 3.12 Nitrite oxidation/anammox: nitrite:nitrate oxidoreductase (*nxrA*)

The community members performing nitrite oxidation in the Bothnian Sea were mainly dominated by anammox bacteria and representatives of the genus *Nitrospira* with low fractions of *Nitrospina*‐related genes detected in the two upper sediment samples. The *nxrA*‐like sequences were detected throughout the whole sediment transect with very similar overall abundance at OAZ (39) and SMTZ (41). Also, the phylogenetic distribution of the *nxrA* reads was very similar in both zones with a clear dominance of anammox (24.6 vs. 25.3) followed by *Nitrospira* (9.7 vs. 9.6). In contrast, the deeper methanic zone harbored significantly less *nxrA*‐like sequences (4), most of which were assigned to *Nitrospira* (2.2) and anammox (1.4). *Nitrospina*‐related *nrc* were at 1.5 in OAZ and 2.7 in SMTZ.

Phylogenetically, Nxr is divided into two only distantly related phylogenetic groups: anammox‐*Nitrospira‐Nitrospina* and *Nitrobacter‐Nitrococccus‐Nitrolancea* (Mogollón, Mewes, & Kasten, 2016). The blast analysis revealed that in all depths nitrifiers from the *Nitrobacter‐Nitrococcus‐Nitrolancea* group did not comprise a significant proportion of *nxrA* reads (4% in each sample).

Previous studies on the ecology and substrate affinities of nitrite‐oxidizing bacteria (NOB) in different environments have pointed to niche differentiation between *Nitrospira*‐ and *Nitrobacter*‐like organisms, which was mainly attributed to available concentrations of nitrite (Bonaglia et al., 2016; Javanaud et al., 2011). Thus, natural ecosystems with limiting nitrite concentrations like the Bothnian Sea sediment would not favor NOB of the *Nitrobacter/Nitrococcus* type.

It has been shown previously that marine environments are mainly dominated by NOB of the genus *Nitrospina* (Bonaglia et al., 2016). Their low abundance in the Bothnian Sea sediment could possibly be explained by the low salinity and brackish conditions. The high abundance of *Nitrospira* was not surprising as its dominance as the main functional NOB has been shown previously for various environments (Conley & Johnstone, 1995). This ubiquity has also partly been attributed to the metabolic flexibility of *Nitrospira* bacteria (Deutsch et al., 2010). The phylogenetic assignment of anammox *nxrA* revealed a diverse community with top blast hits assigned to various freshwater and marine species. The similarity on the amino acid level, however, did never exceed 85% which is in accordance with findings about other genes involved in the anammox metabolism. The Bothnian Sea sediment possibly harbors a new anammox genus.

### 3.13 Dissimilatory ammonia‐forming nitrite reductase (*nrfA*)

*nrfA*‐like gene reads were detected in all depths of the sediment transect, however, their abundance was considerably lower than of those involved in denitrification. Also, more *nrfA*‐like gene fragments were detected in SMTZ (16) than in OAZ (12). The most abundant groups possessing *nrfA* in OAZ were assigned to *Desulfuromonadales* (2), *Bacteroidetes*/*Chlorobi* (3), and *Verrucomicrobia* (1.2). In SMTZ, *Bacteroidetes*/*Chlorobi* (4.1) and *Desulfuromonadales* (3.3) still comprised the most abundant *nrfA*‐possessing groups. Also in MZ most of the *nrfA*‐like reads were assigned to *Bacteroidetes*/*Chlorobi* (3.3). Thus, the *nrfA*‐bearing *Verrucomicrobia* detected in OAZ might be adapted to higher sediment redox state and probably able to tolerate some oxygen.

Previous studies investigating DNRA in estuarine environments reported its relative importance in comparison to denitrification in organic carbon‐ and sulfide‐rich sediments, speculating on inhibitory role of sulfide on denitrification (An & Gardner, 2002). Moreover, it has been speculated that salinity might play a crucial role for the fate of nitrate reduction pathway, where denitrification was inhibited at higher salinities while DNRA was not affected (Giblin, Weston, Banta, Tucker, & Hopkinson, 2010). Based on those previous observations, the combination of low‐sulfide, oligotrophic and hyposaline conditions in the Bothnian Sea sediment would likely favor denitrification over DNRA for nitrate reduction. In fact, the low overall abundance of *nrfA* in comparison to denitrification‐related gene reads supported this hypothesis.

Reports on *nrfA*‐bearing communities in sediments are scarce. So far, these have been analyzed in three estuary ecosystems exhibiting gradients in organic carbon, sulfide, and salinity parameters (Smith, Nedwell, Dong, & Osborn, 2007; Song, Lisa, & Tobias, 2014; Takeuchi, 2006). The majority of *nrfA* sequences detected in the Colne estuary, United Kingdom, were comprised by representatives of δ‐Proteobacteria most closely related to order *Desulfuromonadales*. Despite the very different biogeochemical properties of our sampling site in the Bothnian Sea with the hypernutrified sediment of the Colne estuary, we observed a similar trend in DNRA community toward the dominance of those particular δ‐proteobacterial groups.

### 3.14 Anammox: Hydrazine synthase (*hzsA*)

Metagenome analysis revealed higher *hzsA* gene read abundance in SMTZ (2.4) than in OAZ (0.6). These results contradicted the assumption of anammox bacteria being more abundant in layers where they would have access to oxidized nitrogen oxides for respiration, and corroborated findings on other anammox‐specific genes in the analyzed core. Comparing the identity of *hzsA* reads between both depths, a clear difference was observed. Whereas all reads found in OAZ were assigned to *Scalindua* spp., all reads but one in SMTZ were assigned to *Kuenenia* spp. as top blast hit. Only one read in SMTZ was assigned to *Scalindua*. No *hzsA* gene reads could be identified in MZ. Also the majority of 16S rRNA reads assigned to *Brocadiales* from SMTZ were affiliated with *Kuenenia* and other freshwater anammox genera supporting the findings at the *hzsA* gene level. Despite low read numbers assigned to *hzsA* gene, these results pointed to a vertical stratification of the anammox community within the Bothnian Sea sediment transect. Findings of brackish sediments inhabited by anammox bacteria belonging to different genera including *Scalindua* have been reported before (Dale et al., 2009; Dang et al., 2010a; Dang, et al. 2013b). However, shifts in community structure between freshwater and marine genera have been investigated in horizontal gradients and attributed to changing environmental parameters like salinity, pH or C/N ratio (Dale et al., 2009; Dang et al., 2010a). Slight changes in pH, nitrate/nitrite availability, C/N ratio, or interactions with different metabolic partners between OAZ and SMTZ might be responsible for observed vertical anammox community structure in the Bothnian Sea sediment at site US5B. Also, due to its geographical location the Bothnian Sea is strongly influenced by riverine input from mainland and occasional intrusions of saltier waters from the North Sea. This might have contributed to introduction and preservation of microbial communities from other locations including freshwater habitats of the mainland.

PCR on extracted total DNA with primer pair combinations targeting either the *Scalindua* genus or other five known genera of anammox bacteria resulted in positive amplification only for *Scalindua*‐specific *hzsA* gene. Positive amplification was observed for three samples between 0 and 7.5 cmbsf indicating significant presence of *Scalindua*‐specific *hzsA* genes only in this upper layer. The absence on *hzsA* below 7.5 cmbsf is congruent with metagenome results for MZ where no reads could be assigned to *hzsA* and *Brocadiales*‐specific 16S rRNA genes. However, as PCR product concentration was too low for ligation, a seminested PCR reaction was performed with *Scalindua*‐specific primers for greater yield and further cloning procedure resulting in a final amplicon sequence length of 229nt. In total, 60 amplicon sequences could be retrieved: 21 for 0–2.5 cmbsf, 18 for 2.5–5 cmbsf, and 21 for 5–7.5 cmbsf. All sequences shared 97%–100% amino acid identity with uncultured *Scalindua* spp. originating from the marine sediments in Guyamas Basin (AGV76990) (Russ et al., 2013). However, due to short sequence length, the uncertainty in correct phylogenetic annotation is high and solid information can only be deduced at the genus level.

Biogeochemical parameters of the sediment transect showed measurable nitrate only within the upper 0.5 cmbsf, below this depth the sediment was anoxic. Thus, it remains unclear where an abundant anammox bacterial population would derive nitrogen oxides for respiration below 2.5 cmbsf. The presence of other genes involved in aerobic processes (*amoA*) below 2.5 cmbsf might point to occasional fluxes of both oxygen and nitrogen oxides which would be rapidly consumed thus keeping concentrations below detection limits. An alternative explanation could be the presence of a dormant, not active community which was preserved during the sedimentation process. To our knowledge, deep bioturbation (below 2 cmbsf) which would introduce oxygen in deeper layers was not occurring at the US5B sampling site (Matthias Egger, personal communication).

## 4 CONCLUSIONS AND OUTLOOK

The results of our study indicate the importance of nitrogen cycling in the upper more oxidized Bothnian Sea sediment layers, where, based on genomic potential, full denitrification to N_2_ dominated the N‐cycle driving processes. These findings corroborate previous activity‐based studies showing the dominant role of denitrification and only minor anammox or DNRA. Nitrification was dominated by aerobic ammonia oxidizing *Thaumarchaeota* and nitrite oxidizers belonging to the *Nitrospira* genus, pointing to a similar tight connection of denitrification and nitrification observed before in more eutrophic parts of the Baltic Sea (Thureborn et al., 2013). Unexpectedly, the peak of anammox bacterial community was detected below the oxidized layer, in a zone where no oxygen or nitrogen oxides were detectable and biogeochemistry was dominated by anaerobic methane oxidation with sulfate. Moreover, the anammox community composition seemed to be stratified between different layers with a potentially novel genus, which was not detectable by PCR with specific primers. Generally, identified microbial communities reflected well the biogeochemistry of the analyzed core with most abundant members probably being heterotrophs with multiple respiratory capabilities. Our findings also show that gene amplification‐based techniques might lead to underestimation or lack of detection of microbial key players of particular metabolic processes. This also shows that our understanding of microbial metabolic networks in coastal sediments is still far from being complete.

## CONFLICT OF INTEREST

None declared.
