# Supplementary material for: Metagenomic potential for and diversity of N‐cycle driving microorganisms in the Bothnian Sea sediment
Source: Microbiologyopen. 2017 May 23;6(4):e00475. doi: 10.1002/mbo3.475 (PMC5552932; doi:10.1002/mbo3.475)
Supplement: Supplementary file 1 [file MBO3-6-na-s001.docx]

**Supplementary Table 1**: Cutoff bit score ratios (BSR) used in this study for the analysis of diagnostic N-cycle genes in the Bothnian Sea sediment at site US5B

| gene | OAZ (0-2.5 cmbsf) | SMTZ (5-12.5 cmbsf) | MZ (30-35 cmbsf) |
| --- | --- | --- | --- |
| *narG* | 0.6 | 0.6 | 0.6 |
| *napA* | 0.5 | 0.65 | 0.5 |
| *nirS* | 0.53 | 0.64 | 0.55 |
| *nirK* | 0.6 | 0.6 | 0.6 |
| *nor* | 0.4 | 0.4 | 0.4 |
| *nosZ* | 0.6 | 0.6 | 0.6 |
| *nifH* | 0.84 | 0.86 | 0.7 |
| *amoA** | 0.7 | 0.7 | 0.7 |
| *hao* | 0.55 | 0.55 | 0.65 |
| *nxr*** | 0.85 | 0.85 | 0.85 |
| *nrfA**** | - | - | - |
| *hzsA* | 0.8 | 0.7 | - |

*archaeal *amoA*

***Nitrospira*-*Nitrospina*-anammox-like *nxrA* (for *Nitrobacter*-like *nxrA,* *narG* blastx datasets were checked)

***no BSR cutoff was applied as all blastx hits showed similarity to *nrfA*
