# Supplementary material for: Metagenomic potential for and diversity of N‐cycle driving microorganisms in the Bothnian Sea sediment
Source: Microbiologyopen. 2017 May 23;6(4):e00475. doi: 10.1002/mbo3.475 (PMC5552932; doi:10.1002/mbo3.475)
Supplement: Supplementary file 2 [file MBO3-6-na-s002.docx]

|  |  | **SSU rRNA** | **NxrA** | **NarG** | **NapA** | **NirK** | **NirS** | **Nor/**  **Nod** | **NosZ** | **HzsA** | **NrfA** | **AmoA** | **Hao** | **NifH** |
| --- | --- | --- | --- | --- | --- | --- | --- | --- | --- | --- | --- | --- | --- | --- |
| **OAZ (0-2.5 cmbsf)** | **total** | 397.9 | 38.6 | 55.1 | 29.6 | 91.5 | 73.8 | 37.7 | 52.5 | 0.6 | 12.1 | 22.9 | 33.4 | 10.1 |
| Aquificae | Aquificales | 0.5 | 0.0 | 0.1 | 0.5 | 0.0 | 0.9 | 0.0 | 1.8 | 0.0 | 0.0 | 0.0 | 0.0 | 0.0 |
| Deinococcus-Thermus | Thermales | 0.2 | 0.0 | 1.7 | 0.0 | 0.4 | 0.1 | 0.0 | 0.0 | 0.0 | 0.5 | 0.0 | 1.2 | 0.0 |
|  | Deinococcales | 0.0 | 0.0 | 0.0 | 0.0 | 0.7 | 0.0 | 0.0 | 0.0 | 0.0 | 0.0 | 0.0 | 0.0 | 0.0 |
| Bacteriodetes/Chlorobi | Flavobacteriales | 9.1 | 0.0 | 0.0 | 1.9 | 2.6 | 0.0 | 3.4 | 12.0 | 0.0 | 0.3 | 0.0 | 0.0 | 0.0 |
|  | Cytophagales | 2.4 | 0.0 | 0.0 | 0.0 | 1.5 | 0.1 | 2.4 | 3.5 | 0.0 | 0.5 | 0.0 | 0.3 | 0.0 |
|  | Bacteroidales | 1.0 | 0.0 | 0.0 | 0.0 | 0.0 | 0.1 | 0.6 | 0.0 | 0.0 | 0.7 | 0.0 | 0.0 | 0.0 |
|  | Bacteriodetes_Order_II  _Insertae_Sedis | 1.2 | 0.0 | 0.0 | 0.0 | 0.0 | 0.1 | 0.0 | 2.5 | 0.0 | 0.0 | 0.0 | 0.1 | 0.0 |
|  | Sphingobacteriales | 4.9 | 0.0 | 0.0 | 0.0 | 0.7 | 0.3 | 0.1 | 0.5 | 0.0 | 0.0 | 0.0 | 0.0 | 0.0 |
|  | Ignavibacteriales | 1.7 | 0.0 | 0.0 | 0.0 | 0.0 | 0.0 | 0.0 | 1.6 | 0.0 | 1.5 | 0.0 | 0.3 | 0.0 |
| Verrucomicrobia | Spartobacteria | 0.2 | 0.0 | 0.0 | 0.0 | 2.2 | 0.0 | 0.0 | 0.0 | 0.0 | 0.2 | 0.0 | 0.0 | 0.0 |
|  | Opitutales | 1.7 | 0.0 | 0.0 | 0.0 | 0.2 | 0.0 | 0.0 | 0.8 | 0.0 | 1.0 | 0.0 | 1.0 | 0.0 |
| Acidobacteria | Solibacterales | 0.5 | 0.0 | 0.0 | 0.0 | 0.0 | 0.6 | 0.0 | 0.0 | 0.0 | 0.0 | 0.0 | 0.0 | 0.0 |
| Planctomycetes | Planctomycetales | 15.5 | 0.0 | 0.1 | 1.1 | 0.2 | 0.3 | 2.1 | 0.0 | 0.0 | 0.0 | 0.0 | 0.0 | 0.0 |
|  | Brocadiales | 0.7 | 24.6 | 0.0 | 0.0 | 0.4 | 0.0 | 0.0 | 0.0 | 0.6 | 0.0 | 0.0 | 3.9 | 0.0 |
| Actinobacteria | Actinomycetales | 0.0 | 0.0 | 0.2 | 0.1 | 2.2 | 0.0 | 0.0 | 0.0 | 0.0 | 0.0 | 0.0 | 0.0 | 0.0 |
| Chloroflexi | Herpetosiphonales | 0.0 | 0.0 | 0.0 | 0.1 | 0.4 | 0.0 | 0.0 | 0.0 | 0.0 | 0.0 | 0.0 | 0.0 | 0.0 |
|  | Caldilineales | 1.0 | 0.0 | 0.0 | 0.0 | 0.0 | 0.0 | 0.0 | 1.4 | 0.0 | 0.0 | 0.0 | 0.0 | 0.0 |
| Firmicutes | Clostridiales | 2.2 | 0.0 | 0.0 | 0.6 | 0.6 | 0.0 | 0.0 | 0.0 | 0.0 | 0.2 | 0.0 | 0.0 | 0.0 |
|  | Bacillales | 0.8 | 0.0 | 0.3 | 0.0 | 0.6 | 0.0 | 1.0 | 0.0 | 0.0 | 0.0 | 0.0 | 0.0 | 0.0 |
| Gemmatimonadetes | Gemmatimonadales | 3.4 | 0.0 | 0.0 | 0.0 | 1.3 | 0.0 | 0.0 | 0.1 | 0.0 | 0.0 | 0.0 | 0.0 | 0.0 |
| Nitrospina | Nitrospinales | 0.2 | 1.5 | 0.0 | 0.0 | 1.5 | 0.0 | 0.0 | 0.0 | 0.0 | 0.0 | 0.0 | 0.0 | 0.0 |
| Alpha | Rhizobiales | 1.3 | 0.0 | 0.8 | 0.3 | 3.4 | 0.1 | 0.1 | 0.4 | 0.0 | 0.2 | 0.0 | 0.0 | 0.0 |
|  | Rhodobacterales | 2.7 | 0.0 | 0.7 | 0.0 | 1.1 | 2.9 | 0.3 | 0.4 | 0.0 | 0.0 | 0.0 | 0.0 | 0.0 |
|  | Rhodospirillales | 2.0 | 0.0 | 0.5 | 0.1 | 0.0 | 1.6 | 0.0 | 1.3 | 0.0 | 0.0 | 0.0 | 0.7 | 0.0 |
| Beta | Burkholderiales | 9.2 | 0.0 | 2.4 | 0.7 | 2.6 | 2.2 | 2.8 | 0.4 | 0.0 | 0.2 | 0.0 | 0.3 | 0.0 |
|  | Rhodocyclales | 0.7 | 0.0 | 5.3 | 0.6 | 0.4 | 2.6 | 0.3 | 0.6 | 0.0 | 0.0 | 0.0 | 0.0 | 0.3 |
|  | Gallionellales | 0.2 | 0.0 | 0.0 | 0.0 | 0.0 | 1.0 | 0.4 | 0.0 | 0.0 | 0.2 | 0.0 | 0.0 | 0.0 |
|  | Hydrogenophilales | 1.5 | 0.0 | 0.7 | 0.0 | 0.0 | 3.5 | 0.1 | 0.0 | 0.0 | 0.0 | 0.0 | 0.0 | 0.0 |
|  | Nitrosomonadales | 3.4 | 0.0 | 0.0 | 0.0 | 0.4 | 0.0 | 0.0 | 0.0 | 0.0 | 0.0 | 0.0 | 1.0 | 0.0 |
|  | Sulfuricellales | 0.0 | 0.0 | 0.0 | 0.2 | 0.0 | 1.0 | 0.1 | 0.3 | 0.0 | 0.0 | 0.0 | 0.0 | 0.0 |
| Delta | Desulfobacterales | 3.9 | 0.0 | 0.9 | 1.3 | 0.2 | 0.0 | 0.6 | 0.0 | 0.0 | 0.2 | 0.0 | 1.2 | 0.0 |
|  | Desulfuromonadales | 2.2 | 0.0 | 3.5 | 0.7 | 0.4 | 0.0 | 2.2 | 0.0 | 0.0 | 2.0 | 0.0 | 1.3 | 0.0 |
|  | Myxococcales | 4.2 | 0.0 | 0.6 | 0.5 | 1.5 | 0.3 | 2.9 | 1.0 | 0.0 | 0.5 | 0.0 | 2.9 | 0.0 |
|  | Bdellovibrionales | 1.5 | 0.0 | 0.0 | 0.0 | 0.9 | 0.0 | 0.3 | 0.0 | 0.0 | 0.3 | 0.0 | 0.0 | 0.0 |
|  | Syntrophobacterales | 1.7 | 0.0 | 0.0 | 0.0 | 0.0 | 0.0 | 0.1 | 0.0 | 0.0 | 0.2 | 0.0 | 2.4 | 0.0 |
|  | Desulfovibrionales | 0.0 | 0.0 | 0.0 | 0.0 | 0.0 | 0.0 | 0.0 | 0.0 | 0.0 | 0.0 | 0.0 | 1.2 | 0.0 |
| Gamma | Chromatiales | 1.3 | 0.0 | 0.8 | 1.0 | 1.1 | 2.9 | 0.1 | 0.3 | 0.0 | 0.2 | 0.0 | 0.7 | 1.1 |
|  | Enterobacteriales | 5.4 | 0.0 | 0.8 | 0.3 | 0.0 | 0.0 | 0.0 | 0.0 | 0.0 | 0.0 | 0.0 | 0.0 | 0.3 |
|  | Methylococcales | 19.0 | 0.0 | 3.3 | 0.5 | 4.1 | 8.1 | 2.4 | 0.0 | 0.0 | 0.0 | 0.0 | 0.0 | 7.3 |
|  | Oceanospirillales | 8.7 | 0.0 | 1.1 | 0.2 | 1.7 | 4.9 | 0.3 | 0.8 | 0.0 | 0.0 | 0.0 | 0.4 | 0.0 |
|  | Pseudomonadales | 5.2 | 0.0 | 1.3 | 0.3 | 0.9 | 1.2 | 0.4 | 0.3 | 0.0 | 0.0 | 0.0 | 0.0 | 0.3 |
|  | Thiotrichales | 8.4 | 0.0 | 0.5 | 0.4 | 1.1 | 0.3 | 0.6 | 0.0 | 0.0 | 0.0 | 0.0 | 0.0 | 0.0 |
|  | Alteromonadales | 13.1 | 0.0 | 0.6 | 3.9 | 2.6 | 2.4 | 0.8 | 1.4 | 0.0 | 0.7 | 0.0 | 2.3 | 0.3 |
|  | Vibrionales | 3.0 | 0.0 | 0.0 | 1.5 | 0.0 | 0.0 | 0.1 | 0.0 | 0.0 | 0.0 | 0.0 | 2.2 | 0.0 |
|  | Xanthomonadales | 8.7 | 0.0 | 0.3 | 0.0 | 1.9 | 0.0 | 0.0 | 0.0 | 0.0 | 0.0 | 0.0 | 0.0 | 0.0 |
|  | strain_HdN1/M.oxyfera-like | 0.0 | 0.0 | 0.5 | 0.0 | 0.0 | 0.0 | 2.9 | 0.0 | 0.0 | 0.0 | 0.0 | 0.0 | 0.0 |
| Epsilon | Campylobacterales | 16.6 | 0.0 | 0.1 | 4.1 | 0.0 | 1.6 | 2.1 | 1.7 | 0.0 | 0.0 | 0.0 | 0.0 | 0.0 |
| Nitrospira | Nitrospirales | 2.9 | 9.7 | 0.0 | 0.0 | 0.0 | 0.0 | 0.0 | 0.0 | 0.0 | 0.0 | 0.0 | 0.0 | 0.0 |
| Spirochaetes | Spirochaetales | 2.0 | 0.0 | 0.0 | 0.0 | 0.0 | 0.0 | 2.2 | 0.1 | 0.0 | 0.0 | 0.0 | 0.0 | 0.0 |
| Candidate division OP3 | Candidate division OP3 | 2.2 | 0.0 | 9.9 | 0.0 | 0.0 | 0.0 | 0.0 | 0.0 | 0.0 | 0.0 | 0.0 | 1.4 | 0.0 |
| Euryarchaeota | Haloarchaea | 3.4 | 0.0 | 3.4 | 0.0 | 0.0 | 0.0 | 0.0 | 0.0 | 0.0 | 0.0 | 0.0 | 0.0 | 0.0 |
|  | Methanosarcinales | 0.0 | 0.0 | 0.0 | 0.0 | 0.0 | 0.0 | 0.0 | 0.0 | 0.0 | 0.0 | 0.0 | 0.0 | 0.0 |
|  | Methanomicrobiales | 0.0 | 0.0 | 0.0 | 0.0 | 0.0 | 0.0 | 0.0 | 0.0 | 0.0 | 0.0 | 0.0 | 0.0 | 0.0 |
| Thaumarchaeota | Thaumarchaeota | 12.8 | 0.0 | 0.0 | 0.0 | 39.4 | 0.0 | 0.0 | 0.0 | 0.0 | 0.0 | 21.7 | 0.0 | 0.0 |
| Other/uncharacterized | Other/uncharacterized | 203.8 | 2.8 | 14.7 | 8.6 | 12.3 | 34.7 | 5.7 | 19.4 | 0.0 | 2.7 | 1.2 | 8.6 | 0.6 |
|  |  |  |  |  |  |  |  |  |  |  |  |  |  |  |
|  |  | SSU rRNA | NxrA | NarG | NapA | NirK | NirS | Nor/Nod | NosZ | HzsA | NrfA | AmoA | Hao | NifH |
| SMTZ (5-12.5 cmbsf) | total | 344.6 | 40.5 | 33.9 | 12.4 | 68.2 | 52.9 | 28.9 | 40.4 | 2.4 | 16.2 | 8.9 | 52.0 | 6.1 |
| Aquificae | Aquificales | 0.0 | 0.0 | 0.1 | 0.3 | 0.0 | 0.8 | 0.0 | 0.4 | 0.0 | 0.0 | 0.0 | 0.0 | 0.0 |
| Deinococcus-Thermus | Thermales | 0.0 | 0.0 | 1.8 | 0.0 | 0.4 | 0.0 | 0.0 | 0.0 | 0.0 | 0.0 | 0.0 | 1.4 | 0.0 |
|  | Deinococcales | 0.0 | 0.0 | 0.0 | 0.0 | 1.2 | 0.0 | 0.0 | 0.0 | 0.0 | 0.0 | 0.0 | 0.0 | 0.0 |
| Bacteriodetes/Chlorobi | Flavobacteriales | 6.3 | 0.0 | 0.0 | 0.4 | 0.6 | 0.0 | 4.0 | 7.1 | 0.0 | 0.2 | 0.0 | 0.3 | 0.0 |
|  | Cytophagales | 2.0 | 0.0 | 0.0 | 0.0 | 0.6 | 0.0 | 2.0 | 2.6 | 0.0 | 0.0 | 0.0 | 0.5 | 0.0 |
|  | Bacteroidales | 1.8 | 0.0 | 0.0 | 0.0 | 0.0 | 0.0 | 1.1 | 0.0 | 0.0 | 1.1 | 0.0 | 0.0 | 0.0 |
|  | Bacteriodetes_Order_II_Insertae_Sedis | 0.2 | 0.0 | 0.0 | 0.2 | 0.0 | 0.6 | 0.0 | 2.4 | 0.0 | 0.0 | 0.0 | 0.0 | 0.0 |
|  | Sphingobacteriales | 3.7 | 0.0 | 0.0 | 0.0 | 0.8 | 0.0 | 0.6 | 1.1 | 0.0 | 0.0 | 0.0 | 0.2 | 0.0 |
|  | Ignavibacteriales | 3.3 | 0.0 | 0.0 | 0.0 | 0.0 | 0.0 | 0.0 | 2.1 | 0.0 | 2.8 | 0.0 | 2.1 | 0.0 |
| Verrucomicrobia | Spartobacteria | 0.4 | 0.0 | 0.0 | 0.0 | 0.8 | 0.0 | 0.0 | 0.0 | 0.0 | 0.2 | 0.0 | 0.0 | 0.0 |
|  | Opitutales | 0.2 | 0.0 | 0.0 | 0.0 | 0.0 | 0.2 | 0.0 | 0.6 | 0.0 | 0.2 | 0.0 | 0.5 | 0.0 |
| Acidobacteria | Solibacterales | 0.2 | 0.0 | 0.0 | 0.0 | 0.0 | 0.2 | 1.2 | 0.0 | 0.0 | 0.0 | 0.0 | 0.0 | 0.0 |
| Planctomycetes | Planctomycetales | 32.8 | 0.0 | 0.0 | 1.1 | 0.2 | 0.2 | 1.2 | 0.0 | 0.0 | 0.0 | 0.0 | 0.0 | 0.0 |
|  | Brocadiales | 57.9 | 25.3 | 0.0 | 0.0 | 0.0 | 0.0 | 0.0 | 0.0 | 2.4 | 0.0 | 0.0 | 8.9 | 0.0 |
| Actinobacteria | Actinomycetales | 0.0 | 0.0 | 0.3 | 0.1 | 7.0 | 0.0 | 0.0 | 0.0 | 0.0 | 0.0 | 0.0 | 0.0 | 0.0 |
| Chloroflexi | Herpetosiphonales | 0.0 | 0.0 | 0.0 | 0.0 | 1.4 | 0.0 | 0.0 | 0.0 | 0.0 | 0.0 | 0.0 | 0.0 | 0.0 |
|  | Caldilineales | 3.1 | 0.0 | 0.0 | 0.0 | 0.0 | 0.0 | 0.0 | 1.1 | 0.0 | 0.0 | 0.0 | 0.0 | 0.0 |
| Firmicutes | Clostridiales | 3.1 | 0.0 | 0.2 | 0.1 | 0.2 | 0.0 | 0.0 | 0.3 | 0.0 | 0.0 | 0.0 | 0.8 | 0.3 |
|  | Bacillales | 0.0 | 0.0 | 0.3 | 0.0 | 0.2 | 0.0 | 0.5 | 0.3 | 0.0 | 0.0 | 0.0 | 0.0 | 0.0 |
| Gemmatimonadetes | Gemmatimonadales | 2.6 | 0.0 | 0.0 | 0.0 | 2.3 | 0.0 | 0.2 | 0.4 | 0.0 | 0.0 | 0.0 | 0.0 | 0.0 |
| Nitrospina | Nitrospinales | 0.0 | 2.7 | 0.0 | 0.0 | 0.6 | 0.0 | 0.0 | 0.0 | 0.0 | 0.0 | 0.0 | 0.0 | 0.0 |
| Alpha | Rhizobiales | 3.1 | 0.0 | 0.9 | 0.1 | 2.7 | 0.5 | 0.2 | 0.0 | 0.0 | 0.0 | 0.0 | 0.0 | 0.0 |
|  | Rhodobacterales | 3.0 | 0.0 | 0.3 | 0.1 | 0.6 | 2.7 | 0.0 | 0.4 | 0.0 | 0.0 | 0.0 | 0.0 | 0.0 |
|  | Rhodospirillales | 2.2 | 0.0 | 0.0 | 0.3 | 0.2 | 1.1 | 0.0 | 0.4 | 0.0 | 0.0 | 0.0 | 0.3 | 0.0 |
| Beta | Burkholderiales | 1.1 | 0.0 | 0.9 | 1.1 | 1.0 | 2.7 | 0.2 | 0.7 | 0.0 | 0.7 | 0.0 | 0.2 | 0.0 |
|  | Rhodocyclales | 0.4 | 0.0 | 4.7 | 0.0 | 0.2 | 1.3 | 0.2 | 1.3 | 0.0 | 0.2 | 0.0 | 0.5 | 0.0 |
|  | Gallionellales | 0.0 | 0.0 | 0.0 | 0.0 | 0.0 | 1.4 | 0.5 | 0.0 | 0.0 | 0.2 | 0.0 | 0.2 | 0.0 |
|  | Hydrogenophilales | 0.9 | 0.0 | 0.0 | 0.0 | 0.0 | 4.6 | 0.0 | 0.0 | 0.0 | 0.0 | 0.0 | 0.0 | 0.0 |
|  | Nitrosomonadales | 3.1 | 0.0 | 0.0 | 0.0 | 0.8 | 0.0 | 0.0 | 0.0 | 0.0 | 0.0 | 0.0 | 0.9 | 0.0 |
|  | Sulfuricellales | 0.0 | 0.0 | 0.0 | 0.1 | 0.0 | 1.1 | 0.0 | 0.3 | 0.0 | 0.0 | 0.0 | 0.0 | 0.0 |
| Delta | Desulfobacterales | 13.8 | 0.0 | 2.3 | 0.9 | 0.0 | 0.0 | 1.4 | 0.0 | 0.0 | 0.0 | 0.0 | 8.2 | 0.3 |
|  | Desulfuromonadales | 1.1 | 0.0 | 1.1 | 0.9 | 0.4 | 0.0 | 4.0 | 0.0 | 0.0 | 3.3 | 0.0 | 1.9 | 0.0 |
|  | Myxococcales | 1.1 | 0.0 | 0.3 | 0.4 | 1.4 | 0.2 | 1.4 | 1.8 | 0.0 | 1.3 | 0.0 | 4.1 | 0.0 |
|  | Bdellovibrionales | 0.2 | 0.0 | 0.0 | 0.0 | 0.0 | 0.0 | 0.2 | 0.0 | 0.0 | 0.4 | 0.0 | 0.0 | 0.0 |
|  | Syntrophobacterales | 0.9 | 0.0 | 0.0 | 0.0 | 0.2 | 0.0 | 1.2 | 0.1 | 0.0 | 0.0 | 0.0 | 1.6 | 0.0 |
|  | Desulfovibrionales | 0.2 | 0.0 | 0.0 | 0.2 | 0.6 | 0.0 | 0.2 | 0.0 | 0.0 | 1.3 | 0.0 | 2.4 | 0.3 |
| Gamma | Chromatiales | 0.6 | 0.0 | 0.3 | 0.1 | 0.4 | 1.7 | 0.0 | 0.1 | 0.0 | 0.0 | 0.0 | 0.8 | 0.0 |
|  | Enterobacteriales | 0.9 | 0.0 | 0.2 | 0.1 | 0.0 | 0.0 | 0.0 | 0.0 | 0.0 | 0.0 | 0.0 | 0.0 | 0.0 |
|  | Methylococcales | 0.0 | 0.0 | 0.2 | 0.0 | 0.0 | 0.3 | 0.8 | 0.0 | 0.0 | 0.0 | 0.0 | 0.0 | 0.0 |
|  | Oceanospirillales | 3.7 | 0.0 | 0.0 | 0.1 | 0.4 | 4.1 | 0.0 | 0.3 | 0.0 | 0.0 | 0.0 | 0.5 | 0.0 |
|  | Pseudomonadales | 2.6 | 0.0 | 0.0 | 0.3 | 0.0 | 0.9 | 0.0 | 0.0 | 0.0 | 0.0 | 0.0 | 0.0 | 0.0 |
|  | Thiotrichales | 0.4 | 0.0 | 0.3 | 0.0 | 0.0 | 0.6 | 0.0 | 0.0 | 0.0 | 0.0 | 0.0 | 0.0 | 0.0 |
|  | Alteromonadales | 3.3 | 0.0 | 0.2 | 0.4 | 1.2 | 0.6 | 0.2 | 0.3 | 0.0 | 0.4 | 0.0 | 2.4 | 0.0 |
|  | Vibrionales | 1.1 | 0.0 | 0.0 | 0.0 | 0.2 | 0.0 | 0.0 | 0.1 | 0.0 | 0.2 | 0.0 | 0.9 | 0.0 |
|  | Xanthomonadales | 7.6 | 0.0 | 0.0 | 0.0 | 2.5 | 0.0 | 0.2 | 0.0 | 0.0 | 0.0 | 0.0 | 0.0 | 0.0 |
|  | strain_HdN1/M.oxyfera-like | 0.0 | 0.0 | 0.2 | 0.0 | 0.0 | 0.0 | 2.2 | 0.0 | 0.0 | 0.0 | 0.0 | 0.0 | 0.0 |
| Epsilon | Campylobacterales | 0.7 | 0.0 | 0.1 | 1.1 | 0.0 | 0.2 | 0.0 | 0.4 | 0.0 | 0.0 | 0.0 | 0.2 | 0.0 |
| Nitrospira | Nitrospirales | 3.1 | 9.6 | 0.0 | 0.0 | 0.0 | 0.0 | 0.0 | 0.0 | 0.0 | 0.0 | 0.0 | 0.0 | 0.3 |
| Spirochaetes | Spirochaetales | 4.4 | 0.0 | 0.0 | 0.0 | 0.0 | 0.0 | 0.0 | 0.1 | 0.0 | 0.0 | 0.0 | 0.0 | 0.0 |
| Candidate division OP3 | Candidate division OP3 | 2.2 | 0.0 | 3.6 | 0.0 | 0.0 | 0.0 | 0.0 | 0.0 | 0.0 | 0.0 | 0.0 | 1.3 | 0.0 |
| Euryarchaeota | Haloarchaea | 5.9 | 0.0 | 2.9 | 0.1 | 0.0 | 0.0 | 0.0 | 0.0 | 0.0 | 0.0 | 0.0 | 0.0 | 0.0 |
|  | Methanosarcinales | 4.4 | 0.0 | 0.0 | 0.1 | 0.0 | 0.0 | 0.0 | 0.0 | 0.0 | 0.0 | 0.0 | 0.0 | 3.1 |
|  | Methanomicrobiales | 0.0 | 0.0 | 0.0 | 0.0 | 0.0 | 0.0 | 0.0 | 0.0 | 0.0 | 0.0 | 0.0 | 0.0 | 0.0 |
| Thaumarchaeota | Thaumarchaeota | 5.7 | 0.0 | 0.0 | 0.0 | 11.3 | 0.0 | 0.2 | 0.0 | 0.0 | 0.0 | 8.9 | 0.0 | 0.0 |
| Other/uncharacterized | Other/uncharacterized | 149.2 | 2.9 | 12.8 | 3.4 | 27.7 | 27.0 | 5.5 | 15.5 | 0.0 | 3.9 | 0.0 | 11.2 | 1.8 |
|  |  |  |  |  |  |  |  |  |  |  |  |  |  |  |
|  |  | SSU rRNA | NxrA | NarG | NapA | NirK | NirS | Nor/Nod | NosZ | HzsA | NrfA | AmoA | Hao | NifH |
| MZ (30-35 cmbsf) | total | 318.1 | 3.8 | 3.2 | 0.7 | 23.9 | 4.6 | 5.0 | 7.5 | 0.0 | 4.8 | 6.2 | 6.4 | 1.7 |
| Aquificae | Aquificales | 0.2 | 0.0 | 0.0 | 0.0 | 0.0 | 0.0 | 0.0 | 0.3 | 0.0 | 0.0 | 0.0 | 0.0 | 0.0 |
| Deinococcus-Thermus | Thermales | 0.0 | 0.0 | 0.1 | 0.0 | 0.0 | 0.0 | 0.0 | 0.0 | 0.0 | 0.0 | 0.0 | 0.4 | 0.0 |
|  | Deinococcales | 0.0 | 0.0 | 0.0 | 0.0 | 0.0 | 0.0 | 0.0 | 0.0 | 0.0 | 0.0 | 0.0 | 0.0 | 0.0 |
| Bacteriodetes/Chlorobi | Flavobacteriales | 5.6 | 0.0 | 0.0 | 0.0 | 0.0 | 0.0 | 0.9 | 2.1 | 0.0 | 0.4 | 0.0 | 0.0 | 0.0 |
|  | Cytophagales | 0.6 | 0.0 | 0.0 | 0.0 | 0.2 | 0.0 | 0.3 | 0.6 | 0.0 | 0.0 | 0.0 | 0.0 | 0.0 |
|  | Bacteroidales | 0.0 | 0.0 | 0.0 | 0.0 | 0.0 | 0.0 | 0.5 | 0.0 | 0.0 | 0.8 | 0.0 | 0.0 | 0.0 |
|  | Bacteriodetes_Order_II_Insertae_Sedis | 0.2 | 0.0 | 0.0 | 0.1 | 0.0 | 0.0 | 0.0 | 0.2 | 0.0 | 0.0 | 0.0 | 0.0 | 0.0 |
|  | Sphingobacteriales | 1.9 | 0.0 | 0.0 | 0.0 | 0.0 | 0.0 | 0.0 | 0.3 | 0.0 | 0.0 | 0.0 | 0.0 | 0.0 |
|  | Ignavibacteriales | 4.5 | 0.0 | 0.0 | 0.0 | 0.0 | 0.0 | 0.0 | 0.8 | 0.0 | 2.1 | 0.0 | 0.7 | 0.0 |
| Verrucomicrobia | Spartobacteria | 1.0 | 0.0 | 0.0 | 0.0 | 0.0 | 0.0 | 0.0 | 0.0 | 0.0 | 0.0 | 0.0 | 0.0 | 0.0 |
|  | Opitutales | 0.0 | 0.0 | 0.0 | 0.0 | 0.0 | 0.0 | 0.0 | 0.0 | 0.0 | 0.0 | 0.0 | 0.0 | 0.0 |
| Acidobacteria | Solibacterales | 0.0 | 0.0 | 0.0 | 0.0 | 0.0 | 0.0 | 0.0 | 0.0 | 0.0 | 0.0 | 0.0 | 0.0 | 0.0 |
| Planctomycetes | Planctomycetales | 16.5 | 0.0 | 0.0 | 0.0 | 0.0 | 0.0 | 0.0 | 0.0 | 0.0 | 0.0 | 0.0 | 0.0 | 0.0 |
|  | Brocadiales | 0.0 | 1.4 | 0.0 | 0.0 | 0.0 | 0.0 | 0.0 | 0.0 | 0.0 | 0.0 | 0.0 | 0.7 | 0.0 |
| Actinobacteria | Actinomycetales | 0.2 | 0.0 | 0.0 | 0.0 | 0.0 | 0.0 | 0.0 | 0.0 | 0.0 | 0.0 | 0.0 | 0.0 | 0.0 |
| Chloroflexi | Herpetosiphonales | 0.0 | 0.0 | 0.0 | 0.0 | 0.0 | 0.0 | 0.0 | 0.0 | 0.0 | 0.0 | 0.0 | 0.0 | 0.0 |
|  | Caldilineales | 1.2 | 0.0 | 0.0 | 0.0 | 0.0 | 0.0 | 0.0 | 0.2 | 0.0 | 0.0 | 0.0 | 0.0 | 0.0 |
| Firmicutes | Clostridiales | 4.8 | 0.0 | 0.0 | 0.0 | 0.0 | 0.0 | 0.0 | 0.0 | 0.0 | 0.0 | 0.0 | 0.0 | 0.0 |
|  | Bacillales | 0.2 | 0.0 | 0.0 | 0.0 | 0.5 | 0.0 | 0.0 | 0.0 | 0.0 | 0.0 | 0.0 | 0.0 | 0.0 |
| Gemmatimonadetes | Gemmatimonadales | 0.8 | 0.0 | 0.0 | 0.0 | 0.0 | 0.0 | 0.0 | 0.0 | 0.0 | 0.0 | 0.0 | 0.0 | 0.0 |
| Nitrospina | Nitrospinales | 0.2 | 0.0 | 0.0 | 0.0 | 0.2 | 0.0 | 0.0 | 0.0 | 0.0 | 0.0 | 0.0 | 0.0 | 0.0 |
| Alpha | Rhizobiales | 3.1 | 0.0 | 0.1 | 0.0 | 0.5 | 0.0 | 0.0 | 0.2 | 0.0 | 0.0 | 0.0 | 0.0 | 0.0 |
|  | Rhodobacterales | 1.9 | 0.0 | 0.1 | 0.0 | 0.0 | 0.2 | 0.0 | 0.0 | 0.0 | 0.0 | 0.0 | 0.0 | 0.0 |
|  | Rhodospirillales | 1.7 | 0.0 | 0.0 | 0.0 | 0.0 | 0.0 | 0.0 | 0.0 | 0.0 | 0.0 | 0.0 | 0.0 | 0.0 |
| Beta | Burkholderiales | 1.9 | 0.0 | 0.0 | 0.0 | 0.0 | 0.0 | 0.0 | 0.0 | 0.0 | 0.0 | 0.0 | 0.0 | 0.0 |
|  | Rhodocyclales | 0.6 | 0.0 | 0.0 | 0.0 | 0.0 | 0.4 | 0.0 | 0.0 | 0.0 | 0.0 | 0.0 | 0.0 | 0.0 |
|  | Gallionellales | 0.0 | 0.0 | 0.0 | 0.0 | 0.0 | 0.0 | 0.0 | 0.0 | 0.0 | 0.0 | 0.0 | 0.0 | 0.0 |
|  | Hydrogenophilales | 0.8 | 0.0 | 0.0 | 0.0 | 0.0 | 0.2 | 0.0 | 0.0 | 0.0 | 0.0 | 0.0 | 0.0 | 0.0 |
|  | Nitrosomonadales | 1.4 | 0.0 | 0.0 | 0.0 | 0.2 | 0.0 | 0.0 | 0.0 | 0.0 | 0.0 | 0.0 | 0.5 | 0.0 |
|  | Sulfuricellales | 0.0 | 0.0 | 0.0 | 0.0 | 0.0 | 0.0 | 0.0 | 0.0 | 0.0 | 0.0 | 0.0 | 0.0 | 0.0 |
| Delta | Desulfobacterales | 3.9 | 0.0 | 0.1 | 0.0 | 0.0 | 0.0 | 0.9 | 0.0 | 0.0 | 0.0 | 0.0 | 0.7 | 0.0 |
|  | Desulfuromonadales | 0.6 | 0.0 | 0.1 | 0.1 | 0.0 | 0.0 | 0.5 | 0.0 | 0.0 | 0.4 | 0.0 | 0.2 | 0.0 |
|  | Myxococcales | 2.7 | 0.0 | 0.0 | 0.0 | 0.0 | 0.0 | 0.2 | 0.2 | 0.0 | 0.0 | 0.0 | 0.2 | 0.0 |
|  | Bdellovibrionales | 0.2 | 0.0 | 0.0 | 0.0 | 0.0 | 0.0 | 0.0 | 0.0 | 0.0 | 0.0 | 0.0 | 0.0 | 0.0 |
|  | Syntrophobacterales | 5.8 | 0.0 | 0.0 | 0.2 | 0.0 | 0.0 | 0.0 | 0.0 | 0.0 | 0.0 | 0.0 | 0.2 | 0.0 |
|  | Desulfovibrionales | 0.0 | 0.0 | 0.0 | 0.0 | 0.0 | 0.0 | 0.0 | 0.0 | 0.0 | 0.0 | 0.0 | 0.2 | 0.0 |
| Gamma | Chromatiales | 1.4 | 0.0 | 0.2 | 0.0 | 0.0 | 0.4 | 0.0 | 0.0 | 0.0 | 0.0 | 0.0 | 0.0 | 0.0 |
|  | Enterobacteriales | 0.2 | 0.0 | 0.1 | 0.0 | 0.0 | 0.0 | 0.0 | 0.0 | 0.0 | 0.0 | 0.0 | 0.0 | 0.0 |
|  | Methylococcales | 0.2 | 0.0 | 0.0 | 0.0 | 0.0 | 0.0 | 0.0 | 0.0 | 0.0 | 0.0 | 0.0 | 0.0 | 0.0 |
|  | Oceanospirillales | 2.3 | 0.0 | 0.0 | 0.0 | 0.0 | 0.2 | 0.2 | 0.0 | 0.0 | 0.0 | 0.0 | 0.0 | 0.0 |
|  | Pseudomonadales | 3.7 | 0.0 | 0.0 | 0.0 | 0.0 | 0.0 | 0.0 | 0.0 | 0.0 | 0.0 | 0.0 | 0.0 | 0.0 |
|  | Thiotrichales | 0.6 | 0.0 | 0.0 | 0.0 | 0.0 | 0.2 | 0.0 | 0.2 | 0.0 | 0.0 | 0.0 | 0.0 | 0.0 |
|  | Alteromonadales | 3.1 | 0.0 | 0.0 | 0.2 | 0.0 | 0.0 | 0.0 | 0.0 | 0.0 | 0.0 | 0.0 | 0.4 | 0.0 |
|  | Vibrionales | 0.6 | 0.0 | 0.0 | 0.0 | 0.0 | 0.0 | 0.0 | 0.2 | 0.0 | 0.0 | 0.0 | 0.4 | 0.0 |
|  | Xanthomonadales | 9.3 | 0.0 | 0.0 | 0.0 | 0.0 | 0.0 | 0.5 | 0.0 | 0.0 | 0.0 | 0.0 | 0.0 | 0.0 |
|  | strain_HdN1/M.oxyfera-like | 0.0 | 0.0 | 0.0 | 0.0 | 0.0 | 0.0 | 0.0 | 0.0 | 0.0 | 0.0 | 0.0 | 0.0 | 0.0 |
| Epsilon | Campylobacterales | 0.0 | 0.0 | 0.0 | 0.0 | 0.0 | 0.0 | 0.0 | 0.0 | 0.0 | 0.0 | 0.0 | 0.0 | 0.0 |
| Nitrospira | Nitrospirales | 3.3 | 2.2 | 0.0 | 0.0 | 0.0 | 0.0 | 0.0 | 0.0 | 0.0 | 0.0 | 0.0 | 0.0 | 0.0 |
| Spirochaetes | Spirochaetales | 11.0 | 0.0 | 0.0 | 0.0 | 0.0 | 0.0 | 0.0 | 0.0 | 0.0 | 0.0 | 0.0 | 0.0 | 0.0 |
| Candidate division OP3 | Candidate division OP3 | 5.4 | 0.0 | 0.9 | 0.0 | 0.0 | 0.0 | 0.0 | 0.0 | 0.0 | 0.0 | 0.0 | 0.5 | 0.0 |
| Euryarchaeota | Haloarchaea | 24.4 | 0.0 | 0.3 | 0.0 | 0.0 | 0.0 | 0.0 | 0.0 | 0.0 | 0.0 | 0.0 | 0.0 | 0.0 |
|  | Methanosarcinales | 5.0 | 0.0 | 0.0 | 0.0 | 0.0 | 0.0 | 0.0 | 0.0 | 0.0 | 0.0 | 0.0 | 0.0 | 0.7 |
|  | Methanomicrobiales | 1.0 | 0.0 | 0.0 | 0.0 | 0.0 | 0.0 | 0.0 | 0.0 | 0.0 | 0.0 | 0.0 | 0.0 | 0.3 |
| Thaumarchaeota | Thaumarchaeota | 21.5 | 0.0 | 0.0 | 0.0 | 14.5 | 0.0 | 0.2 | 0.0 | 0.0 | 0.0 | 5.2 | 0.0 | 0.0 |
| Other/uncharacterized | Other/uncharacterized | 162.4 | 0.3 | 1.2 | 0.0 | 7.8 | 3.2 | 0.9 | 2.4 | 0.0 | 1.0 | 1.0 | 1.4 | 0.7 |

Supplementary table 2: Normalized read values (nrc) for diagnostic N-cycle genes and the corresponding 16S rRNA genes in the Bothnian Sea sediment at site US5B.
